# Mr. Key's Cases of Femoral Hernia and Impenetrable Stricture

**Published:** 1835-04-01

**Authors:** 


II. *Operation for Femoral Hernia, without opening the Sac.*

Ann Hunt, æt. forty-two, was admitted at Guy's Hospital, on the 4th of January last, suffering under strangulated hernia. Her complexion was florid, and she stated her general health to be very good; she said that she was married, and the mother of six children, the last of which was born several years ago. The hernia, which was femoral, was of sixteen months standing; during which time it had always been returned with facility, and retained by a truss. About forty-eight hours before her admission, having removed her truss, the hernia protruded, and very soon she became affected with pain in the part and sickness. A medical man was called in, who merely sent her medicine for the alleviation of the sickness, as she had concealed from him at first the fact of her being ruptured. When he became acquainted with it, he endeavoured to reduce the hernia; but, failing to do so, he sent her to the hospital. The hernia was then extremely hard and painful to the touch; her abdomen was tumid and tender, and her bowels had not been relieved for fifty hours; the sickness constant during the same period. Pulse 120, small; tongue brown, and furred. She was put into the warm bath, and the taxis employed for about fifteen minutes, and again for a short time, when the bath had produced syncope, but without success.

Mr. Key now saw her, and, finding the tumour still unyielding, she was removed to her bed, and the operation commenced in the usual manner. After dividing a portion of the fascia propria, a lobulated mass presented itself, very much like omentum, but consisting of fat brought down by the hernia from the crural opening. Through this Mr. Key introduced the flat director he employs in this operation, and, having divided the stricture external to the sac, the slightest pressure on the part sufficed to return the bowel to the abdomen. Her symptoms were immediately relieved, and two hours after the operation she complained of nothing but slight smarting in the wound. She was ordered.—℞. Magnes. Calc. ℈j.; Magnes. Sulph. ʒij. statim sum. Injiciatur Enema c. Ol. Ricini ℥ss. post horam. This acted twice on her bowels in about four hours. Pulse, ninety-six.

On the following day, January 5th; her countenance was exceedingly good, tongue clean, skin moist, pulse eighty-eight. She had but little sleep. Bowels not opened.—Rep. Mist. 3tia quaque hora, si op. fuerit.

6th. Bowels opened twice in the night. She slept well; tongue clean; pulse seventy-six.

7th. The same.

8th. The medicine occasioned flatulence, and was suspended.

9th. She complained of much pain in the lower part of the abdomen; skin hot; tongue white; pulse 108. She had pain on inspiration.—Hirudines XL. abdomini. Calom. gr. ij.; Opii, gr. j. 6ta quaque hora.

10th. Pain much relieved; passed a tolerably good night; tongue less furred; pulse eighty; bowels not opened.—Enema c. Ol. Ricini ℥ss.

12th. Towards evening she had a return of the pain in the abdomen; tongue furred; pulse ninety-six.—Hirudines XL. Rep. Pil.

13th. Better: much relieved by the leeches; skin moist. —Cont. Pil.

14th. Much better. She has had a swelling in the right iliac fossa, but which is now much smaller.

16th. Going on well. The swelling in the side scarcely perceptible.

18th. She complained of acid eructations; for which, a few small doses of Potassæ Carb. were administered. After this she had not a bad symptom. The wound healed on the 30th, and she was discharged.

III. *Impenetrable Stricture, with Urinary Fistula. Operation.*

Samuel Huie, æt. thirty. Eight years ago, while engaged as a planter in Jamaica, he fell across a beam, and injured his perineum: the accident was followed by retention of urine and extravasation. A small catheter was introduced thirty-six hours after the fall, and was kept in the bladder two days; the integuments and urethra had then sloughed, and laid open the passage several inches. It remained so twelve months, when the edges of the wound were pared, and ligatures applied. He wore a catheter till this had healed, which was in about two months. When it was withdrawn, the urethra became strictured at the same point, and at length almost impervious; only admitting the stilette of an elastic catheter. From this time he frequently suffered from retention of urine, and relieved himself by the introduction of the wire. About twelve months since, a small abscess formed in the perineum, behind the stricture, which burst, and the urine has been passed in part by the opening up to the present time. He was taken into the hospital, October 8th. No instrument could be passed through the stricture, except the small stilette, which he introduced with considerable dexterity. The urethra, at the termination of the bulb, was very hard and irregular, and behind it was a small opening in the perineum, which would scarcely admit the smallest probe. Micturition was attended with great pain; the urine passing guttatim from the fistulous opening, and in a minute stream, about the size of a horsehair, from the penis.

After he had been in the house some weeks, Mr. Key, finding it impossible to cure the stricture by dilatation, proposed an operation, similar to the one which had before been performed, viz. that of opening the urethra with a knife, and allowing it to heal over a catheter. This was done on the 10th of November. The quilled suture was used, but removed three days afterwards, in consequence of a small slough appearing between the edges of the wound. A week after the operation, the catheter was withdrawn, cleaned, and returned without difficulty. On the 10th of December, the opening was very small: Mr. Key introduced a smaller catheter, and ordered the Ung. Hyd. Nit. Ox. to be introduced on lint, with a probe.

On January 1st, the wound in the perineum was closed, except a very minute opening. The catheter was withdrawn, and ordered to be introduced, when the patient wished to pass urine. The wound healed on the 12th; he introduced the catheter himself with facility, and was recommended to do so for some time, when he found it requisite. He left the hospital on the 17th.

